# The decision-making in dribbling: a video analysis study of U10 soccer players’ skills and coaches’ quality evaluation

**DOI:** 10.3389/fpsyg.2023.1200208

**Published:** 2023-07-24

**Authors:** Enzo Iuliano, Valerio Bonavolontà, Dafne Ferrari, Nicola Bragazzi, Benito Capasso, Goran Kuvačić, Andrea De Giorgio

**Affiliations:** ^1^Faculty of Psychology, eCampus University, Milano, Italy; ^2^Department of Applied Clinical and Biotechnological Sciences, University of L’Aquila, L'Aquila, Italy; ^3^Department Unicusano, Niccolò Cusano University, Rome, Italy; ^4^Laboratory for Industrial and Applied Mathematics (LIAM), Department of Mathematics and Statistics, York University, Toronto, ON, Canada; ^5^Faculty of Kinesiology, University of Split, Split, Croatia; ^6^Klinikos Center for Psychodiagnostics and Psychotherapy, Rome, Italy

**Keywords:** youth soccer, performance, skills, quali-quantitative analysis, football

## Abstract

**Introduction:**

Dribbling is an important soccer skill that, when effective, allows players to overcome opponents. It can provide a strong tactical advantage; for this reason, all of its components (sprint, speed, and ball control) are fundamental to the development of young players. Dribbling can also be considered a decision-making process, and due to its characteristics, it is not always easy to study ecologically. Using a video analysis study, this research aimed to determine whether dribbling skills, specifically dribbling choice (i.e., decision-making), were related to U10 soccer players’ quality.

**Methods:**

Several outcomes measures, divided into three categories, were taken during video analyses: (i) measures related to the efficacy of dribbling skill; (ii) measures related to the ability of players without the ball to support the player in possession; and (iii) measures related to ball circulation. These data were retrospectively assessed to whether the coaches had formed the teams in training through an implicit knowledge of the players’ dribbling skills.

**Results:**

The percentage of accurate dribbling (that is, the ability to perform correct passes after a successful dribble) was found to be the variable that coaches may have implicitly used in creating the three groups differentiated by technical skills (*p* < 0.05). In fact, this percentage was 12.9%, 24.0%, and 48.1% for the groups with lower, average, and higher technical skills, respectively.

**Conclusion:**

Overall, the results demonstrate that dribbling accuracy has an important weight in the coach’s evaluation of the technical skills level of young soccer players.

## 1. Introduction

Soccer is considered an open-skill sport in which players have to react to unpredictable external stimuli, such as decisions and movements both of the opponent and teammate. Among the numerous actions that can be performed in soccer, dribbling – a technique also used in other sports such as basketball or handball, where a player moves the ball by bouncing it while running or walking – is the most frequently executed as it leads to a greater tactical advantage (Reilly et al., 2000), and its effectiveness is a hallmark of more skilled players (Ali, 2011).

Due to its characteristics, and when performed effectively, dribbling is a crucial soccer skill that allows players to overcome opponents. For these reasons, the components of dribbling (sprint, speed, and ball control) are fundamental in the development (Huijgen et al., 2009, 2010) and training of young players (e.g., De Giorgio et al., 2018). In a longitudinal study by Huijgen et al. (2009), it was shown that talented youth players who became professionals were better at dribbling performances than those who remained amateurs. The authors concluded that dribbling performance during adolescence could be a reliable predictor of senior success in soccer. Russell et al. (2011) showed and discussed that speed and precision are essential when assessing dribbling efficacy in elite youth soccer players. Still, dribbling efficacy must be evaluated before accuracy and speed. Zago et al. (2016) investigated the determinants of effective dribbling skills through a motion capture system in a group of ten sub-elite under−13 players. Their investigation showed that to improve dribbling efficacy, it is crucial to develop and design specific practices that require high stride frequency and narrow run trajectories, because faster players can run with the ball through a shorter path in a more economical way. So far, investigations into dribbling efficacy have mainly focused on kinematics (e.g., Gioldasis et al., 2022), physical characteristics (e.g., Gidu et al., 2022), disabilities (e.g., Chénier et al., 2022), and biomechanics (e.g., Chen et al., 2021).

To the best of the authors’ knowledge, no studies have determined the relationship between dribbling and passing possibilities granted by its eventual success. Additionally, it was shown that dribbling decisions in futsal were influenced by the diversity of passing angles, shooting, and especially interpersonal distance, whose variability affected dribbling success (Corrêa et al., 2016). The possibilities offered before and after dribbling can be encompassed in a cognitive process called decision-making (Moutoussis et al., 2021), although in the field of sport, it is preferable to refer to it as naturalistic decision making (NDM; see, e.g., Macquet and Fleurance, 2007; Krstulović et al., 2021; Bossard et al., 2022). Indeed, soccer is a sport in which action is not regulated by an *a priori* plan, and players choose whether to dribble against the opponent based on different characteristics present at a given time (i.e., naturalistic).

Given the naturalistic decision-making characteristics, studying it in soccer is very complex. Soccer players’ decisions, such as whether to dribble or pass the ball when facing an opponent, were inferred *via* video analysis of an actual practice match to conduct naturalistic research. Furthermore, because coaches choose the players who play according to their experience, this study was retrospective in nature to assess whether there was a correspondence between coaches‘choice to form three teams based on players‘skills and the effectiveness of soccer players‘dribbling belonging to each team. Therefore, this study aimed to determine whether dribbling skills, specifically dribbling choice (i.e., decision making), were related to the players’ quality.

## 2. Materials and methods

### 2.1. Experimental design

This empirical study used descriptive observational analysis with an arbitrary observation code, where the phenomenon was produced in the habitual natural environment without researcher intervention (Montero and León, 2007; Seif-Barghi et al., 2012). The participants were divided into three groups with an equal number of participants (12 participants in each group) based on their basic technical soccer skills (reception, passing, ball management, and shooting), but soccer players were unaware of how they were assigned to groups. The formation of the three teams was done by the coaches according to their judgment of the players’ quality: the group with higher technical skills (HIGH); the group with average technical skills (AVERAGE); and the group with lower technical skills (LOW). The coaches involved had: (i) Master’s Degree in Sports Science; (ii) UEFA C license; and (iii) four years of experience in the same club.

Players were utterly blind to their skill level determined by the coach to avoid a situation where someone’s high expectations tend to improve a person’s performance, the so-called Pygmalion effect (Weinstein et al., 1987). Finally, each group was randomly divided into two teams composed of 6 players each (e.g., HIGH group was divided into team 1 and team 2). The study was performed in the winter period. During this time, participants had an average training volume of 3 training sessions per week. Their training routine was monitored and controlled by two professional soccer coaches.

### 2.2. Participants

Thirty-six nine-year-old soccer players (height: 136.6 ± 5.1 cm; body mass: 35.6 ± 2.5 kg) from Italian U10 soccer schools voluntarily participated in this study. The following inclusion criteria were adopted: (i) players had to be in good health with no presence of cardiovascular disease, illness, injury, pain, and metabolic syndrome symptoms; (ii) to have a signed sports medical certification by a sports medicine specialist; and (iii) to have at least 1 year of soccer training. After being thoroughly informed about experimental procedures, guardians gave their written informed consent to participate in the study as the sample consisted of underage individuals. Each participant had the full right to withdraw from the study at any time during the testing. Ethical Committee of the University of Split, Faculty of Kinesiology (number: 2181-205-02-05-22-0026) approved all procedures that were in accordance with the 1975 Declaration of Helsinki ethical principles for scientific investigations involving human participants.

### 2.3. Variables

Several outcome measures divided into three categories were taken during video analyses (Table 1 shows all measures with descriptions):

1. Measures related to the dribbling efficacy skill: (i) the number of successful dribbling; (ii) the number of unsuccessful dribbling; (iii) the number of total dribbling; (iv) the number of successful dribbling with the correct pass (accurate dribbling); (v) the number of successful dribbling with a wrong pass (inaccurate dribbling); and (vi) the total number of no useful dribbling (unsuccessful dribbling + inaccurate dribbling).
2. Measures related to the ability of the players without the ball to support the player focused on dribbling: (i) the number of times in which there was a teammate in a supporting position available; and (ii) the number of times in which there was no teammate in supporting position available.
3. Measures related to the ball circulation: (i) the number of passes in the match; (ii) passes/total dribbling ratio; passes/successful dribbling ratio; and (iii) passes/accurate dribbling ratio.

**Table 1:** Description of the outcome measures.

Variable		Description
Dribbling	Succesfull	Total number of dribbling performed by all the players of a team during the match in which the player successfully overcame a player of the opposite team
	Unsuccessful	Total number of dribbling performed by all the players of a team during the match in which the player did not overcome a player of the opposite team
Dribbling	Accurate	Total number of correct passes performed after a successful dribbling by all the players that were useful to create an advantageous situation for the team
	Not Useful	Total number of wrong passes performed after a successful dribbling by all the players that did not allow to create an advantageous situation for the team despite the positive outcome of the dribbling AND total number of Unsuccessful dribbling
Successfull dribbling	Accurate	Same that accurate dribbling
	Inaccurate	Total number of wrong passes performed after a successful dribbling by all the players that did not allow to create an advantageous situation for the team despite the positive outcome of the dribbling
Supporting position	Yes	Number of times in which at least one teammate was available to support the player that was dribbling during all the match
	No	Number of times in which no teammate was available to support the player that was dribbling
Number of	Total dribbling	Total number of dribbling performed by all the players of a team during the match regardless of their outcome
	Passes in the match	Total number of passes performed by all the player of a team during the match
Ratio	Passes/Total dribbling	Ratio between the number of passes in the match and the total dribbling
	Passes/Successful dribbling	Ratio between the number of passes in the match and the successful dribbling
	Passes/Accurate passes	Ratio between the number of passes in the match and the accurate dribbling

**Figure 1:**
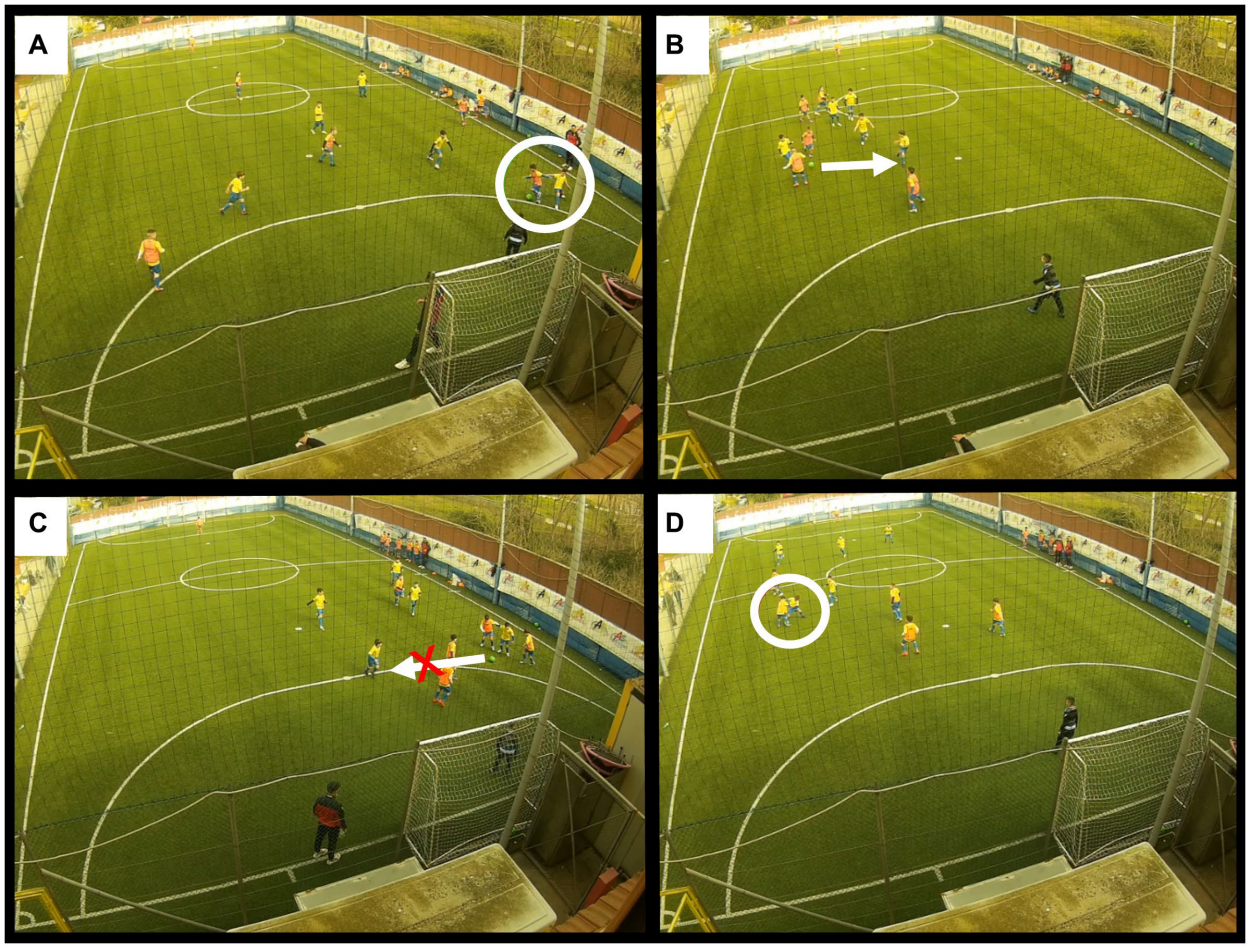
Conditions related to dribbling: **(A)** successful dribbling performed by a child wearing an orange bib; **(B)** successful dribbling with passing option performed by a child wearing a yellow t-shirt; **(C)** successful dribbling without passing option performed by a child wearing the yellow t-shirt; and **(D)** unsuccessful dribbling tried by a child wearing an orange bib.

We want to emphasize that dribbling is considered here not for its quality, but regarding its successful/unsuccessful. Indeed, when a footballer has an opponent in front of him, he can make three decisions: shoot on goal, pass the ball, or dribble. The dribbling followed by a direct shot on goal was not considered due to their very low numerosity (4 in total for the three matches). For these reasons, a score of 1 was assigned every time the player performed a successful dribbling and 0 when it was unsuccessful. Figure 1 highlights the exact moments when dribbling was evaluated under the conditions already mentioned. These conditions were evaluated by two other coaches who were unaware of the study’s purpose: they were asked to judge whether a dribbling was successful or unsuccessful, whether there were passing opportunities, and whether the pass made was correct or incorrect. The analysis in our study was therefore based on the judgments made by two coaches, i.e., when their assessments matched. Video analysis was performed using the Oumox SJ5000 Camera (Qumox Ltd., Hong Kong, China). The camera was positioned above one of the two goals at a height of 9 meters above the ground to record players. The recorded videos were analyzed using LongoMatch v. 1.7 software (Fluendo, SA, Barcelona, Spain).

### 2.4. Procedures

Participants played three matches on three different days according to the following schedule: day 1: HIGH group (team 1 vs. team 2); day 2: AVERAGE group (team 1 vs. team 2); day 3: LOW group (team 1 vs. team 2). Each match consisted of three 15 min periods according to federal rules in the U10 category.^1^ Matches were played with a ball size four on a 30 × 15 meters soccer field with synthetic turf. The goals measured 3 × 2 meters, and an official soccer referee was recruited to ensure the fairness of the matches. During the days that preceded the matches, players were not involved in a heavy training routine to avoid any fatigue interference on the testing. Additionally, to eliminate any circadian variation, all matches took place at the same time (between 5 pm and 6.30 pm) and under the same climate conditions (clear weather, 10 ± 3°C temperature, and 31 ± 1.7% relative humidity). The principal investigator carried out all procedures, gave instructions, explained the procedures, and monitored matches. The second investigator recorded matches, and the third controlled the testing procedure conditions.

### 2.5. Statistical analysis

Due to the nature of the data, non-parametric statistical analyses were used to test the hypothesis of the study. All the variables were described using relative (%) and absolute frequencies (*n*). After the descriptive analysis was performed, an inferential analysis was performed to compare groups. Association between the variables was estimated using the Chi-square (*χ*^2^) tests. Due to its ability to calculate a more exact value of *p* with smaller sample sizes, Fisher’s exact test (FET) was applied over the Chi-square test of independence (Portney and Watkins, 2015). The Adjusted Standardized Residuals (ASR) from the contingency tables (Williams and Wragg, 2006) were analyzed to determine whether the observed cell frequency statistically differed from the expected cell frequency. The ASR is superior to unadjusted standardized residuals as it considers the number of comparisons and the sample size and reports a more accurate difference. The observed frequency is significantly greater than the expected frequency if the ASR is greater than 1.96 (*p* < 0.05). In contrast, the observed frequency is significantly less than expected if the ASR is less than −1.96 (*p* < 0.05). The strength of association between variables was estimated Cramer’s V (V_c_) with the following four ranges according to Crewson (2006): small (values <0.100), low (values between 0.100 and 0.299), moderate (values between 0.300 and 0.499), and high (values >0.500). The significance level was set at *p* < 0.05. All data were analyzed with SPSS 28.0 statistical software (SPSS, Chicago, IL, United States) and GraphPad Prism 9 (GraphPad Software, Inc., San Diego, CA, United States).

## 3. Results

**Figure 2:**
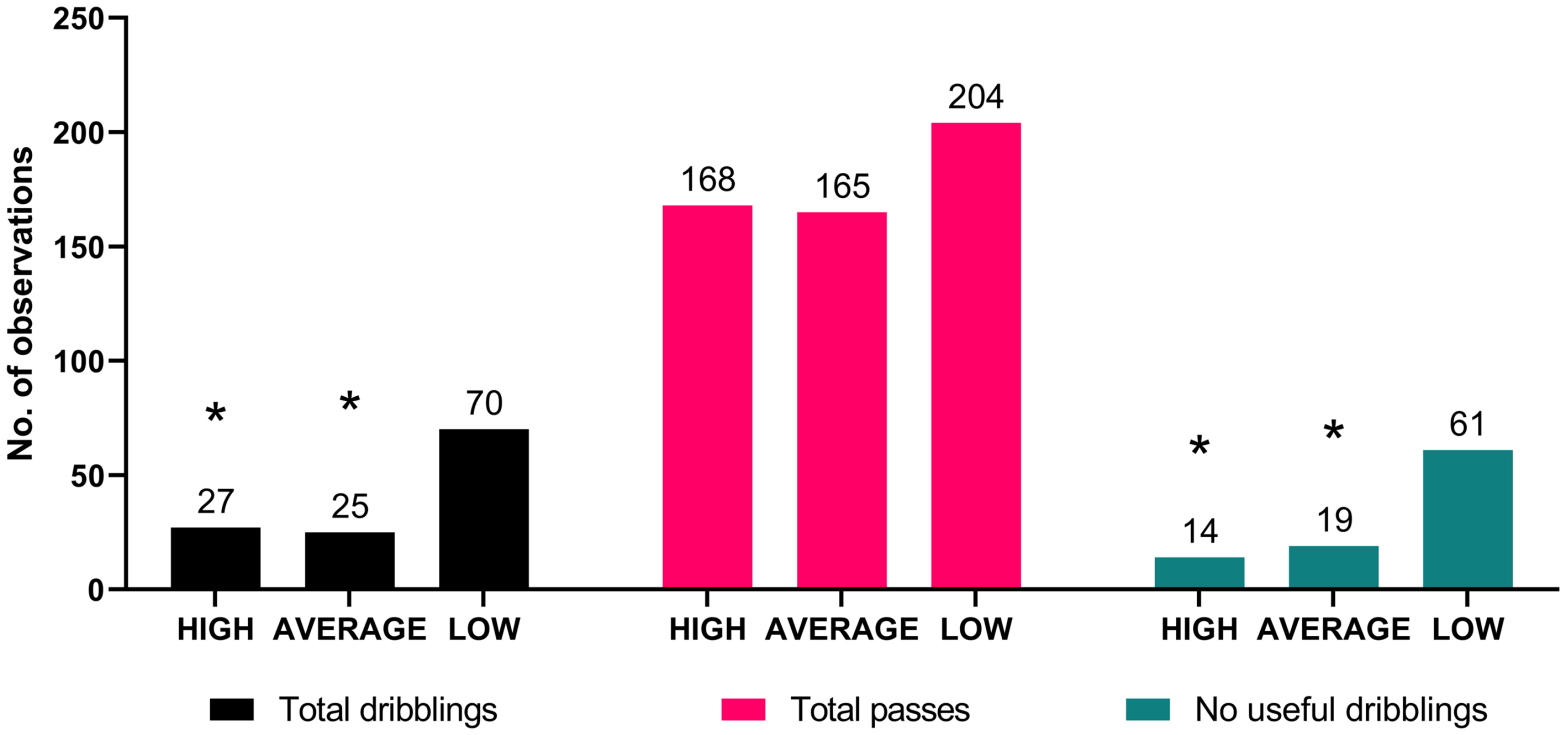
Associations between groups in total passes, dribbling, and no useful dribbling. HIGH – higher basic technical skills group; AVERAGE – average technical skills group; LOW – lower technical skills group; * significantly lower than LOW group at *p* < 0.001.

Figure 2 presents associations between groups in total passes, dribbling, and no useful dribbling observed during matches. There were fewer observations for the HIGH and AVERAGE groups and more observations for the LOW group in the number of dribbling [*χ*^2^(2) = 31.79, *p* < 0.001] and no useful dribbling [*χ*^2^(2) = 42.53, *p* < 0.001]. Total passes were equally distributed across all groups [*χ*^2^(2) = 5.26, *p* = 0.07].

Table 2 shows descriptive analysis and the ASR of the categories of each variable in all groups. A significant relationship has been found between groups in successful/unsuccessful dribbling during the match [FET = 6.318, *p* = 0.04, V_c_ = 0.23 (low)]. The ASR indicated that the HIGH group, compared to the AVERAGE and LOW groups, had more successful dribbling than expected. Moreover, the highest number of not useful dribbling was observed in the LOW group, while The HIGH group had the lowest [FET = 12.78, *p* < 0.01, Vc = 0.37 (moderate)]. Similarly, according to the ASR values, the HIGH group had significantly more accurate dribbling (correct passes) when compared to the AVERAGE and LOW groups [FET = 8.13, *p* < 0.05, V_c_ = 0.37 (moderate)]. In contrast, The LOW group was far less successful in accurate dribbling performance. There was no significant relationship between groups in the supporting position, meaning that all groups performed as expected [FET = 1.13, *p* = 0.58, V_c_ = 0.1 (low)].

**Table 2:** Associations between groups in dribbling efficacy, supporting position, and ball circulation.

Variable		HIGH (*n* = 12)			AVERAGE (*n* = 12)			LOW (*n* = 12)		
		*n*	%	ASR	*n*	%	ASR	*n*	%	ASR
Dribbling	Successful	19	70.4	2.5*	10	40.0	-1	31	44.3	−1.3
	Unsuccessful	8	29.6	−2.5*	15	60.0	1	39	55.7	1.3
Successful dribbling	Accurate	13	68.4	2.3*	6	60.0	0.9	9	29.0	−2.8*
	Inaccurate	6	31.6	−2.3*	4	40.0	−0.9	22	71.0	2.8*
Dribbling	Accurate	13	48.1	3.5*	6	24.0	0.1	9	12.9	−3.1*
	Not useful	14	51.9	−3.5*	19	76.0	−0.1	61	87.1	3.1*
Supporting position	Yes	6	22.2	0.4	3	12.0	−1.1	15	21.4	0.6
	No	21	77.8	−0.4	22	88.0	1.1	55	78.6	−0.6
Ratio	Passes/Total dribbling	6.2			6.6			2.9		
	Passes/Successful dribbling	8.8			16.5			6.6		
	Passes/Accurate passes	12.9			27.5			22.7		

ASR, adjusted standardized residuals; * residuals > 1.96 significantly greater than the expected frequency or residuals < −1.96 significantly less than the expected (*p* < 0.05); HIGH – higher basic technical skills group; AVERAGE – average technical skills group; LOW – lower technical skills group.

## 4. Discussion

To the best of our knowledge and based on a recent systematic review (Aquino et al., 2017) this is the first study that attempts to determine if the coaches‘experience can predict the quality of soccer players based on their decision-making skills in dribbling.

The data obtained from the real training match video and their subsequent analysis showed significant differences among the three teams, confirming our hypothesis that the coach correctly chosen the players to be assigned to the teams based on their dribbling skills. Moreover, the main differences existed between the HIGH and LOW groups. To summarize the overall results and data obtained, it could be affirmed that the HIGH group preferred dribbling quality over quantity, whereas the LOW group preferred dribbling quantity over quality. At the same time, the AVERAGE group seems to be somewhere in the middle.

Indeed, more dribbling was not useful since this did not increase their effectiveness. In other words, the players of the HIGH group seem to have chosen to dribble where they were more confident in success, while the players of LOW tried dribbling much more; from this it is possible only to infer the difficulty in reading the game situation correctly. The data reported in Table 2 showed that the LOW group tried a considerably higher number of dribbling compared with the HIGH and AVERAGE groups (70 vs. 27 and 25, respectively), but only 9 dribbling’s were accurate compared with the 13 accurate dribbling of the HIGH group and the 6 of the AVERAGE group. These data concerning the number of accurate dribbling seems to suggest that the LOW group had better results than the AVERAGE group (9 vs. 6 accurate dribbling’s), but the AVERAGE group tried to perform only 25 dribbling in the match, whereas the LOW group tried 70 dribbling in total.

When the percentage ratio between the number of correct dribbling’s and the total number of dribbling’s is considered (Table 2; Dribbling – Accurate – % values), the three groups have the following percentage values: 12.9% in the LOW group, 24.0% in the AVERAGE group, and 48.1% in the HIGH group. It is therefore evident how the value of this percentage doubles moving from the LOW group to the AVERAGE group and then the HIGH group. In other words, after data analysis, it is possible to infer that the percentage of accurate dribbling’s may be considered one of the implicit variables that coaches use in creating the three groups differentiated by technical skills.

Since there is no literature on this particular topic, it can be inferred that coaches are unaware of this particular variable, except implicitly, as they globally judge the abilities of soccer players. In other words, they tend to choose the best players to form teams without consciously thinking or slavishly calculating in numerical terms – among others – the dribbling skills.

The number of successful dribbling (that does not consider the success in passing the ball to a teammate but only the success in dribbling) and the respective percentage compared to the total number of successful dribbling (Table 2; *Dribbling – Successful – n and % values*), was more favorable to LOW group compared to the AVERAGE group. It can be inferred that implicitly for the coaches, the total number of successful dribbling, as well as the percentage of successful dribbling compared with the total number of dribbling performed, was not so important compared with the ability to create accurate situations by successfully dribbling’s and then efficaciously pass the ball to a teammate.

Dribbling is regarded as one of the most important predictors of success in soccer, and standardized tests to measure this and other soccer skills have been conducted in the literature (Russell et al., 2010). Recently, Konefał et al. (2019) investigated what frequency of technical activity is required to improve results by analyzing an entire domestic season match in Germany‘s Bundesliga. Authors demonstrated that this skill is crucial in soccer players, especially when it comes to maintaining a favorable score. Interestingly, researchers found that dribbling was more effective when the team was already winning and needed to keep the lead, rather than when the draw had to be maintained or was to be achieved. Wilson et al. (2020) found that dribbling speed was related to goal-scoring success in a variety of situations, including curved paths. To better study this ability, the authors concluded that specific training useful for improving dribbling should be conducted. Our findings seem indicate that coaches place a high value on young soccer players’ dribbling accuracy and for this reason it is possible to infer that at an implicit level, and among different soccer skills and variables, they seem to compose the teams according to the dribbling abilities.

Nevertheless, another aspect to take into account is the game field dimension. The present study conducted experiment on a 30 × 15 meters soccer field. Previous studies indicated that the dimension of the game field, as well as the number of players, can influence the technical skills outputs. For example, Aslan (2013) indicated that the number of ball possessions and unsuccessful passes was higher on a small pitch than on a large one in adult soccer players. Other studies on an adult young adult population (17–22 years old) revealed that the technical actions that changed as a consequence of field size changes, were the number of passes, tackles, and shots, which decreased as the field dimension increased (Kelly and Drust, 2009; Hodgson et al., 2014). Concerning the younger players, with age similar to that of the players considered in the present study, the literature lacks investigations on large soccer fields. However, some studies have evaluated the differences between matches performed with a different number of players. Garcia and colleagues (Garcia et al., 2014) discovered that young soccer players (U9 and U14) perform more touches of the ball and attacking plays in the smaller game formats (5 vs. 5 and 7 vs. 7) than in the 9 vs. 9 formats. Katis and Kellis (2009) discovered similar results, observing that the number of short passes, kicks, tackles, dribbling’s, and goals scored in U14 players was significantly higher during the 3 vs. 3 game condition compared to the 6 vs. 6 game condition.

It is possible to conclude how the results obtained by adults and younger players appear to be overall contradictory: in adult players, increasing the ratio player/surface appears to improve technical demand on players, as stated by Hodgson et al. (2014); in younger players, increasing the ratio player/surface appears to induce a decrease in technical demand on players who frequently perform more long passes (Katis and Kellis, 2009).

These results are potentially in accordance with the results of the present study because a possible interpretation could be that players with higher technical skills (overall adult players; HIGH group) prefer to perform technical elements only when they are reasonably sure of the success to avoid unnecessary errors. As a result, soccer players with superior technical skills increase the number of technical elements performed only when the distances between them are short and the match is fast. On the other hand, players with lower technical skills appear to be less concerned with making errors and increase the number of technical elements performed as soon as they have more space available.

It is important to note that, as stated by Roberts et al. (2021): “*Coaches are an integral part of talent identification in sport, and they are often used as the ‘gold standard’ against which scientific methods of talent identification are compared*.” On the other hand, the same authors affirmed that the coaches’ decision-making is not well understood. The present study can be considered as an attempt to objectify what some authors call “gut instinct (Roberts et al., 2021), a particular process that probably focuses on identifying those variables that are considered fundamental for the sport that is practiced. A recent study indicated that dribbling skills, 15-m sprint time, and height are the best variables to discriminate U17 soccer players competing at national level from players competing at regional level (Nughes et al., 2020). However, a direct comparison of results is difficult due to the differences between the methodologies used. Finally, we cannot exclude that a Pygmalion effect may have influenced the players’ performances. Solomon (2001) conducted a study to determine whether coaches could accurately predict their athletes’ achievements based on their expectations. The study found that only the coach’s assessment of the athlete’s self-confidence was a reliable predictor of sports achievement, but no other expectations were related. Moreover, if the coach inaccurately evaluates an athlete’s skills, even talented players may never reach their full potential (Short and Short, 2005). For these reasons, it seems difficult to believe that a player who cannot dribble properly would become able to do so solely due to the Pygmalion effect. Furthermore, it is important to emphasize that the players were not instructed to try to do as many dribbles as possible, but played their games freely, without any mandate regarding the exaggeration of techniques or tactics.

## 5. Conclusion

It is essential to underline that success is not only a product of a player’s physical and technical skills but also related to the scenario. Consequently, players’ skills should not be assessed outside the real context of the game, but they can only be evaluated in real-world scenarios. It can therefore be deduced that the point of view of the coach plays a fundamental role. This study represents an attempt to objectify the variables that can represent the implicit coach opinion. Replicating the present study methodology on a larger number of coaches and teams, as well as more matches, could provide further and important indications on what are the key technical skills of the youth sectors in coaches’ eyes and evaluate whether coaches’ views reflect the effective characteristics required in the current soccer context.

## Data availability statement

The raw data supporting the conclusions of this article will be made available by the authors, without undue reservation.

## Ethics statement

The studies involving human participants were reviewed and approved by Ethical Committee of the University of Split, Faculty of Kinesiology (number: 2181-205-02-05-22-0026). Written informed consent to participate in this study was provided by the participants' legal guardian/next of kin.

## Author contributions

ADG, EI, VB, and BC: conceptualization. GK and BC: methodology. GK and NB: software. EI, VB, DF, NB, BC, GK, and ADG: validation, writing—original draft preparation, and writing—review and editing. BC and DF: formal analysis. EI, ADG, and BC: investigation. GK and EI: data curation. DF: visualization. ADG: supervision. ADG and BC: project administration and funding. All authors contributed to the article and approved the submitted version.

## Conflict of interest

The authors declare that the research was conducted in the absence of any commercial or financial relationships that could be construed as a potential conflict of interest.

## Publisher’s note

All claims expressed in this article are solely those of the authors and do not necessarily represent those of their affiliated organizations, or those of the publisher, the editors and the reviewers. Any product that may be evaluated in this article, or claim that may be made by its manufacturer, is not guaranteed or endorsed by the publisher.

## Footnotes

^1^ https://www.figc.it/media/123300/allegato-2-modalit%C3%A0-di-gioco-categorie-di-base-2020-2021.pdf
